# Cocaine

**Published:** 1896-07

**Authors:** Samuel A. Hopkins

**Affiliations:** Boston, Mass.


### COCAINE.[1]

BY SAMUEL A. HOPKINS, M.D., BOSTON, MASS.

Ever since the discovery of anæsthesia the idea of a local anæsthetic has taken firm hold of the professional mind, and no amount of failure has been able to shake our belief that some day or other the ideal local anæsthetic will be forthcoming. It must be safe, it must be easy to administer, and it must be quick in its effect.

[1] Read before The New York Institute of Stomatology May 5, 1896.

We need a safe local anæsthetic. There is perhaps no greater need in modern surgery and dentistry. The mental anguish, as well as the actual suffering entailed by our daily operations is appalling. It makes the profession of dentistry the most trying and exhausting one known. The pain which we are obliged to give is not only injurious to the health of the patients, but it limits our usefulness, and even affects the estimation in which our profession is held by the community at large. So much, therefore, is to be gained by the use of a local anæsthetic that the temptation is very great to try everything that may promise an alleviation of pain, and we are loath to give up anything that seems even in a moderate degree to give that alleviation, although its use may be attended by grave risks. Indeed, we sometimes deceive ourselves so great is the desire to save pain, and are almost persuaded that any risk is justifiable to gain such an end.

This, I am afraid, has been the history of the use of cocaine in the dental profession. It is a sufficient comment on its doubtful efficacy in dentistry that after so many years of experimentation it should still be necessary to publish articles in our dental journals describing its use and its limitations, and the fact that you are willing to listen to me to-night indicates that grave doubts still exist in your minds as to its unrestricted use in dentistry being justifiable.

In order to get at the facts concerning the use of this drug I have gone over very carefully a very large amount of literature on the subject, and have looked up some of the records of the cases where serious results are reported. I am indebted for valuable information to Dr. T. W. Hays, who, in March, 1894, before the Cincinnati Academy of Medicine, read a most interesting paper on the physiological action of cocaine, citing numerous authorities.

The symptoms of cocaine-poisoning differ materially in different individuals, and there is probably a disposition or diathesis existing in some individuals which renders them exceedingly susceptible to the drug. Great difference exists in regard to the time it takes for poisoning effects to appear. This may vary from thirty seconds to three hours, and the time necessary for recovery also varies greatly. Sometimes recovery is almost immediate, and, on the other hand, it may take months, and then leave the patient in a very anæmic condition. The sex, age, or condition of the patient does not seem to have any particular influence on the effect. Strong or weak, young or old, all may be quickly affected. Neither are habitual users of the drug, those who have formed the cocaine habit, entirely free from the dangers of acute poisoning.

The size of the dose and the method of its administration do not seem to control the effect. There is, however, seemingly a peculiar susceptibility when the drug is applied or injected in the vicinity of the fifth nerve or its branches. Woffler, who favors the use of cocaine, states that in most of the cases that have come under his observation where serious or fatal results have followed its use the injections were made in some part of the head. He claims that a five-per-cent. solution may be used with impunity in other parts of the body, but a solution of not greater strength than two per cent. can safely be used in the region of the head.

In a general way, the symptoms of cocaine-poisoning may be briefly described as follows: There is an excitation of the mind, and associated with it an expression of anxiety which may amount to a fear of approaching death. A feeling of warmth steals over the patient, which may be followed by a chill. The respiration becomes fearfully rapid, and later becomes labored. The pulse-beat increases to a very marked degree, and the pulse runs up to 150, or even higher. Respiration becomes more difficult, and the heart grows weak, while the mental disturbance is increased so that ideas do not follow each other in proper sequence. If the drug has been administered in the mouth, the tongue becomes numb and speech is affected; not always, however, to such a marked degree as might be expected. If the poisonous effects continue, there seems to be more general anæsthesia, and the organs so affected have a decided feeling of cold. Sometimes an irritation along the spine or back of the neck, a tickling or itching sensation, is present. The hands are closed in a convulsive manner; the fingers, legs, and arms become stiff and tetanic. The muscles of the face partake of the convulsive movements, and the expression is agonizing to the last degree. In some cases death occurs while in this tetanic condition. Sometimes, however, instead of the convulsive symptoms complete relaxation takes place. If recovery occurs, severe nervous disturbances may remain for an indefinite time.

The symptoms I have described as belonging to cocaine-poisoning are subject to wide variation. Indeed, no two cases seem to give exactly the same train of symptoms. In going over the records I have been astonished to find how many cases of poisoning are reported. Mannheim reports five cases of poisoning from the subcutaneous use of the drug, and also nine cases in which it was dropped into the eye; two where it was used in the ear; larynx, three; mouth, two; gum, two, etc. Four drops of a two-percent. solution used by injection produced poisoning in an old lady, who did not recover for four days. Three drops of a three-per-cent. solution was followed in one case by marked restlessness, which disappeared in four days. 0.05 grain in one case and 0.04 grain in another injected subcutaneously into the eyelid caused intoxication lasting many hours. Dr. Hays, among other cases, mentions the fact that he himself was poisoned by cocaine injected into the gum. It is but fair to state that the dose was large, but the poisoning was almost instantaneous.

The March number of the *Centralblatt* mentions a fatal case of poisoning following an injection into the urethra. In the October, November, and December, 1890, numbers of *Therapeutische Monatsschrift* is given a complete list of the reported cases of poisoning up to that date. The percentage of fatal cases is enormous. Of one hundred and seventy-six cases recorded, ten were fatal. Enough has been said of the general poisoning effects to show that the drug is one to be used with great caution. We do not yet know what its dangers may prove, nor have we yet found a physiological antidote. Digitalis, atropine, the nitrite of amyl, and nitro-glycerin have been suggested, but the efficacy of these drugs is still a matter of doubt.

I found it a much more difficult matter to get any definite reports of serious results following the use of cocaine in dental practice. This was, of course, to be expected, as most of these cases occur in private practice, and cannot be reported without injury to the reputation of the practitioner. Consequently, we get only meagre accounts of the unsuccessful cases, or of those cases which are attended by fatal or alarming symptoms. Each successful case is, however, quickly reported, much to the glorification of the operator, and the temptation to follow in his footsteps leads to many an accident.

Appreciating this difficulty, I wrote to a number of leading New York dentists, asking for their experience in the use of the drug. I also asked, "What should be considered the maximum dose for hypodermic injection into the tissues of the mouth?" This last question has never been answered, nor can I answer it myself.

The greatest courtesy was shown me, however, and I received a large number of replies to my letter, but I will not attempt to read them, as I have promised to be brief. A large number of those who so kindly answered my letter frankly said they were unwilling to risk hypodermic injections in any strength. A few alluded to the alkaloid, isoatropyl-cocaine, and its dangers, and all expressed the feeling that great caution should be used in administering the drug hypodermically. Dr. Delafield, under whom I had the plêasure of studying at the College of Physicians and Surgeons, wrote that he thought it should never be used hypodermically. My own experience seems to bear out this opinion, so far as it relates to injections into the tissues of the mouth.

I have myself had the scare, and although several years have now passed, it sends a cold chill down my back whenever I recall my experience. One case I shall never forget of a man in apparently perfect physical condition, for whom I had to perform an operation upon the gum between the lower first and second molars, which necessitated giving great pain. My syringe was carefully sterilized, and ten minims of a four-per-cent. solution were taken into the syringe; two minims remained after the operation, and at least two minims were absorbed by the napkins with which I surrounded the parts, so that it is safe to say that the results following were produced with a dose of not over six minims. The gum was relaxed and somewhat torn, and undoubtedly a part of the dose got into the mouth and may have been swallowed. Be that as it may, in about ten minutes there was a seeming excitation of the mind, and all the symptoms which I have previously described soon followed. I was obliged to get my patient onto a bed, and send for his physician. By the aid of stimulants—brandy and coffee—the patient gradually recovered, and was able to be driven to his home in three or four hours.

In another case, that of a young woman, the patient left the office in apparently good condition after an operation in which the drug had been used, but complained that while in the car on her way home she experienced difficulty in breathing, with palpitation of the heart, and had to exert all the power of her will to get to her house. She did not recover fully for several days. In this case not over four minims of a four-per-cent. solution were used.

A similar case to the above was that of a vigorous young man, who was a student. He likewise did not feel the effects until on his way to Cambridge; but as he had access to several convenient bar-rooms on his way out, he was able to get sufficient strength to reach his rooms. In this case not over three minims of a four-per-cent. solution were injected, and none escaped into the mouth. While the after-effects of this case were not serious, a terror of similar results was produced that made him willing to submit to the most painful operations rather than have me make use of the drug again.

I have found hundreds of cases of cocaine-poisoning reported in various medical journals, both in this country and abroad, and shall be glad to give these references to any one who wishes to investigate this subject further.

Suggestions of new methods for doing away with the danger have appeared from time to time during the past fifteen years, and in the December number of the *Dental Digest* an article from the pen of a careful observer suggests the combination of cocaine with morphine and atropine.

Whether we have any safe or reliable substitute for cocaine in the various combinations that are suggested from time to time has not yet been determined. There is, however, a substance which deserves more than passing notice on account of the high character and scientific standing of the men who have made the investigations concerning it. Dr. A. P. Chadbourne, of Boston, in 1892, before the British Medical Association, read a valuable paper on an alkaloid which had recently been isolated by Giesel from the leaves of a small-leaved cocoa-plant of Java. The chemical constitution and properties of this substance were studied by Liebermann, who proved that it was benzoyl-pseudo-tropein. Chadbourne gave it the name of tropa cocaine, and under that name it is now sold by the leading manufacturers of drugs.

In his paper, a careful study of which I would recommend to any one who contemplates using this drug, Dr. Chadbourne relates a series of carefully-performed experiments with tropa cocaine, using cocaine of equal strength upon the control animals. I cannot, of course, give these experiments in detail, but the conclusions drawn, which were amply supported by the evidence, were as follows:

1. Tropa cocaine is less than one-half as toxic as cocaine.
2. The depressing action both on the cardiac motor ganglion and the heart muscle, especially the latter, is much greater with cocaine.
3. Local anæsthesia, both of the eye and of the skin, is much more complete with tropa cocaine, and is possibly of longer duration.
4. Solutions of tropa cocaine are moderately antiseptic, and retain their strength for at least two or three months, while cocaine solutions begin to deteriorate in as many days.

Experimentation on the human subject confirmed the above conclusions and seemed to demonstrate that tropa cocaine was twice as strong and half as toxic as cocaine. There is, however, one possible source of error in using tropa cocaine,—viz., the possibility of obtaining an impure sample of the drug. Dr. Chadbourne, in his experiments, procured one sample that was much more toxic than the others; not more toxic than cocaine, however. But after purification by recrystallization the difference disappeared.

It is also rather expensive, and the supply of small-leaved coca-plants is naturally limited. One other difference might be considered an objection. Cocaine has a contractile action on the small blood-vessels, which tends to arrest hemorrhage; tropa cocaine has no such action.

After writing the above, I took the liberty of calling upon Dr. Chadbourne, and asked him if he had seen any reason to modify his views on the subject of the two drugs. He assured me that the experience of those who had made use of tropa cocaine only tended to confirm the conclusions he had drawn from his experiments. In the course of our conversation he gave me an important point in the treatment of cocaine-poisoning, which I have not seen referred to elsewhere. He found that with the animals experimented upon a much larger dose of cocaine could be used if the temperature of the room were lowered, and the animal recovered from the toxic effects more quickly when the body temperature was lowered by exposure to cold. This suggests that an ice-pack or exposure to the cold air in winter might give considerable relief in these unfortunate cases of cocaine-poisoning. He also confirmed the statement I have already made that an injection in any part of the head is more liable to be attended by toxic symptoms than in other parts of the body.

I do not wish to be understood as advocating the hypodermic injection of this new drug into the gum for the extraction of teeth, or other operations. I merely wish to affirm that if a local anæsthetic is to be used in this way that there is some scientific basis for experimentation with tropa cocaine, and it will probably be found much more effective and a thousand times safer than any of the nostrums that are offered to the public as substitutes for cocaine.

One other method of local anæsthesia I must refer to, as it is now before the public, supported by men of recognized skill and intelligence,—namely, the use of the electric current in connection with cocaine and guaiacol, or cocaine alone. For a more detailed account I would refer you to Dr. W. J. Morton's article in the January number of the *Dental Cosmos*, to Dr. Gillett's article in the February number of the INTERNATIONAL DENTAL JOURNAL, and to other more recent articles, which will repay careful study.

I have seen cocaine used by cataphoresis a sufficient number of times to convince me that it has a place in dental practice, and I mean to use this method for obtunding sensitive dentine in extreme cases. It is, however, too cumbersome to be used as a routine method. There is usually more or less pain, sometimes a good deal of nervous apprehension attending its use, and in some cases it fails to make any appreciable difference in the sensitiveness of the tooth. This may be due to faulty manipulation. The dam should always be in place when this method is applied, as I am not convinced that cocaine used in this way is less poisonous than when used in the ordinary manner. Two possible improvements have suggested themselves to me,—one would be to have the obtunding done by an assistant, and thus save fifteen minutes of your valuable time, for the loss of time is a serious objection to this process; and the other would be the substitution of tropa cocaine.

The suggestion has recently been made that the incisors may be rendered insensible to the touch of the instrument by placing pellets of cotton saturated with a ten-per-cent. solution of cocaine in the nostrils. I have seen this tried with entire satisfaction, but I hesitate to endorse the method from the danger of forming the cocaine habit. Doubtless, you know that snuffing cocaine up the nose is a particularly delightful form of indulging in the cocaine habit and one that is easily acquired. Singers sometimes acquire the habit by using the drug to dry up the secretions and get temporary relief while singing. Neither the morphine nor the alcohol habit compares with the cocaine habit in the undermining influence on the mind and body. For this reason I should discourage the use of cocaine by this method.

Another method of producing local anæsthesia with cocaine was suggested by Dr. Schleich, of Berlin, who recently published a monograph on the subject. Briefly, it consists of an almost infinite number of injections of an almost infinitesimal amount of the drug. The injections are made, not subcutaneously, but intracutaneously, and the technique is somewhat as follows: Beginning always in the healthy skin, and holding the syringe almost parallel with the skin, the needle is introduced, great care being taken not to push it through the skin. The fluid will distend the skin and raise a white, bloodless wheal. This area is instantly anæsthetic. Keeping within this area, you introduce the needle near its edge and produce another œdematous white spot. In this way you can gradually anæsthetize a foot of territory. The anæsthesia lasts about twenty minutes, and infiltration can be repeated if necessary. In dealing with inflamed tissue it is always desirable to encroach upon it gradually from the surrounding healthy tissue. In operations requiring deep incisions the gradual process should be adopted in getting at the seat of the disease.

The strongest solution used contained only two-tenths of one per cent. of cocaine, and the weak solution contained only one-hundredth of one per cent. of cocaine, with a little salt solution added. Indeed, it is pretty evident that the anæsthesia comes more from pressure on the terminal nerve filaments than from the drug itself, since it can be shown that a two-tenths-per-cent. salt solution injected in the same way will produce anæsthesia; not, however, without severe irritation. Chemists are prepared to furnish tablets made according to the formula of Schleich, and this is, perhaps, the best form in which to obtain the drug for this method of administration. How general this method will become no one can predict, but I know of the successful removal of a good-sized abdominal tumor, several operations for varicocele, the opening of a felon, and a lot of minor operations. The mouth hardly offers the best field for the practice of this method, but in a general way it is not without interest.

I cannot close this paper without a reference to the wholesale extraction of teeth by ignorant or unprincipled practitioners, who advertise the painless extraction of teeth by the use of so-called obtundents. For several years past we have been receiving from time to time in our mails advertisements of obtundents which are to be used by injection. In almost every instance the advertiser claims that no cocaine is used, and tempting offers of exclusive territory and dazzling riches to follow the use of this particular preparation are held out to the unwary. By reference to an article in the May (1893) number of the *Dental Cosmos* it will be seen that nearly all of these preparations contain a large percentage of cocaine. This article is by Dr. Edward C. Kirk, of Philadelphia, who had a number of these so-called local anæsthetics chemically examined in the Philadelphia College of Pharmacy. There were ten different preparations, all of which had been widely advertised, and in almost every case the impression had been given, if it had not been positively stated, that the preparation contained no cocaine. It was found on analysis that every one of the preparations, with the exception of Barr's, which was merely an alcoholic solution of peppermint and cloves, contained cocaine, and many of them in such large amounts as to be dangerous even in small doses.

Unfortunately, the use of these preparations seems to be increasing. Of course, no self-respecting man could be guilty of violating the code of ethics of his profession by manufacturing and advertising such nostrums. It is well understood that the profession has a right to any discovery or improvement that may be made by one of its members, and each man in the profession is under distinct obligation to give to the profession any knowledge that he may have acquired that will benefit his fellow-practitioners.

It seems to me that it is equally a violation of the code to use and recommend any nostrums that may be put upon the market. In this particular case there is an additional reason for taking a high stand, as a disguised danger is more to be dreaded than an open one. It would be well if we could bring about such legislation as would make it a criminal offence to deceive the public by flooding the market with such nostrums, but I am not sanguine about our power to institute reforms by legislative action. We have to combat not only the inertia of political bodies, but the opposition of uneducated and unprincipled practitioners as well. Every peripatetic tooth-puller eagerly avails himself of these preparations, reckless of the danger, and while it would be a great gain to humanity to exterminate this species of dental practitioner, I can see only one way to accomplish it, and that is by a crusade of education and the creation of a higher and better public opinion.

It is the duty of every man in the profession to use his influence in warning the public against these fearful traps laid for the unwary. Every means in our power should be used to expose the charlatans who, for a fee, are willing to subject a patient to any risk, and who are doing irreparable injury by the wholesale extraction of valuable teeth. We must check this evil if we wish to uphold the dignity of our profession and preserve our self-respect. How we can handle this problem best it is difficult to know; but with high ideals and a high appreciation of our calling we can carry on an aggressive warfare that will eventually result in the extermination of nostrum manufacturers and irresponsible practitioners.

